# Cleaning Up Muddy Waters: The Fight to Revive Senegal’s Hann Bay

**DOI:** 10.1289/ehp.124-A92

**Published:** 2016-05-01

**Authors:** Jori Lewis

**Affiliations:** Jori Lewis writes about the environment, agriculture, and international development from her perch in Dakar, Senegal. She is currently writing a book about the early history of peanuts in West Africa.

When PhD student Ibrahima Diagne has to take a sample of the wastewater flowing from Dakar’s drainage canals into Hann Bay, he does the smart thing: Diagne sends the master’s student who came with him to do it. On a recent morning, that student, Cheikh Tidiane Dione, rolled up his pants, tied plastic bags around his hands, and got to work in the cloudy water, careful to avoid the trash that accumulates around the canal’s opening. As he took the sample, a woman came by with a bucket of her kitchen scraps and tossed them straight into the water.

Further down the beach, Dione took a sample from another canal that runs black and thick with something viscous—oil, Diagne hypothesized—combined with fecal matter. In the opposite direction, a different canal releases malodorous wastewater from an industrial zone that includes several food companies and a tannery. The bay receives wastewater from many industrial sources, including chemical companies, an abattoir, and an oil refinery. More than 10 years ago, Marc Bouvy, a researcher with the French Research Institute for Development (IRD), undertook a study of bacterial contamination in the bay.^1^^,^^2^ He says that several pipes from industrial sources released unknown pollutants, including one that “one day released red wastewater and other days released waters that were green and yellow.”

**Figure:**
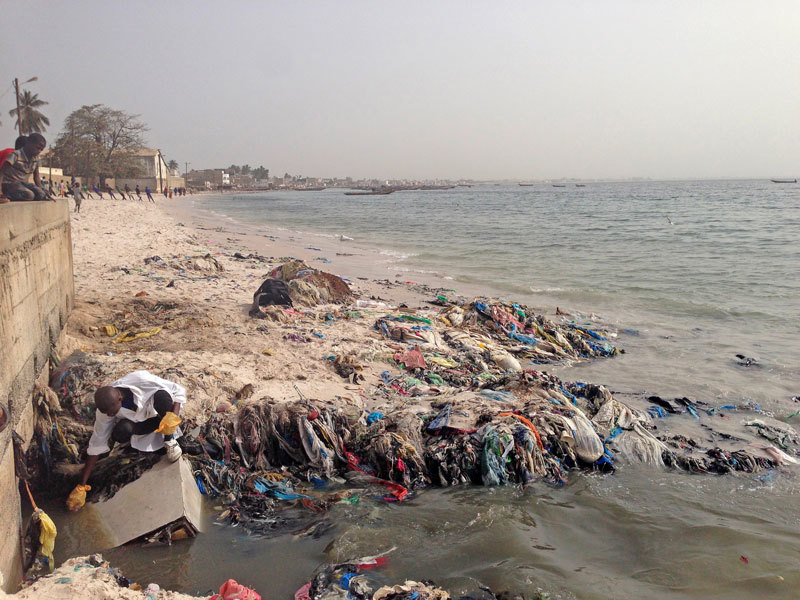
Once a sparkling jewel with white sand beaches, Senegal’s Hann Bay is now severely polluted with untreated sewage, industrial waste, and more. Researchers are studying what’s in the water, how it’s affecting life around the bay, and how it can be cleaned up. Here, student Cheikh Tidiane Dione takes samples of wastewater from one of the canals feeding into the bay. © Jori Lewis

These are just a few of the many outflows that stream into the bay, a stretch of beach that runs from the industrial port of Dakar about eight miles to the outskirts of the city. All of them, the canals and the pipes, have been pouring their waste into the bay unimpeded and untreated for years. Now researchers are filling in details about what’s polluting the waters of the bay and how to mitigate the problem.

## Unknown Health Risks

The calm shores of Hann Bay were once home to miles of pristine white sand beaches, the best in greater Dakar and often compared with Rio de Janeiro’s Copacabana. But the free-flowing emissions from all the industries and homes have combined to transform the area from an idyllic paradise to one of the most polluted waterways in Senegal.

Hann Bay, like all bays, is semi-enclosed and protected from the waves of the open ocean. The bay provided a good place for fishermen to settle hundreds of years ago, and eventually it became a safe place for ships to dock. But the topography of the bay also limits the water’s circulation, compared with the open ocean. So, when wastewater and trash find their way through the canals, they follow the current throughout the bay before moving out into the ocean^1^

According to Bouvy, most of the levels of fecal indicator bacteria he measured in the bay were well above the levels established by the European Union.^1^ Contact with water contaminated with high levels of fecal indicator bacteria is strongly associated with the incidence of respiratory, dermatological, and gastrointestinal problems.^3^^,^^4^^,^^5^^,^^6^

Amary Seck believes the polluted water is responsible for his own respiratory problems. He grew up in one of the communities along the bay, Petit Mbao, and still lives there. “I went to the hospital, and they told me it’s nothing, but personally, I know that something is wrong,” he says. His 5-year-old son, too, has asthma. Amary Seck says that many people in his community have similar problems. But little research has been done to prove the links between health problems and pollution in these communities.

**Figure:**
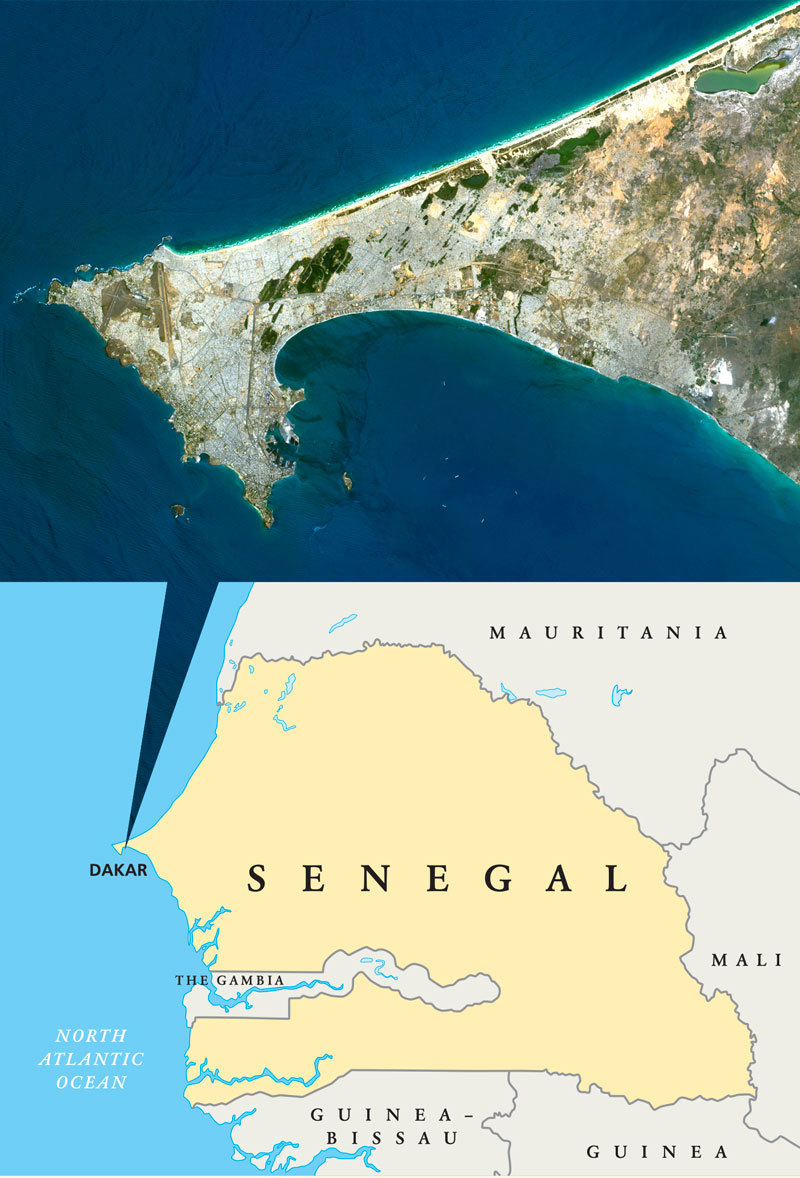
Dark streaks appear in the waters of Hann Bay, which curves around the inside hook of the Cap-Vert Peninsula. Dakar’s population has roughly quintupled since 1960 as migrants from the interior of Senegal fled drought and searched for new economic opportunities. Satellite image: © Planet Observer UIG; map: © Peter Hermes Furian/Shutterstock

The health issues are complex, and the pollution of the bay is almost certainly not the only factor. The government conducted some health studies in recent decades, but they were never published and are not publicly available. According to a 2005 assessment by the United Nations Environment Programme, 35% of the people from one Hann Bay community who were examined by physicians at the university hospital have skin disease, which the doctors attribute to water pollution in the bay.^7^

Ibrahima Diagne, the PhD student, is studying the influence that wastewater from the canals has on the chemical composition of water in the bay. “The level of ammonia is very high in the canal. And even when you go out ten meters into the bay, the level of ammonia remains high, much higher than the seawater,” says Diagne. The levels of sulfates and phosphates are likewise high, and all those excess nutrients contribute to the development of algae, which in turn deplete the water of the oxygen that fish populations need to survive.

## A Breakdown in the System

Anything that limits the survival of fish populations is more than a theoretical concern. Hann is a working bay ringed with fishing communities. In fact, the outflow where Diagne and Dione took samples is on the edge of a large fish market, where fishermen and merchants spread out their hauls of sardines, barracudas, sharks, and eels on the sand to sell. In the distance, nearly a dozen fishermen worked together to haul in a net that was situated far offshore. Pulling from the shore, they strained in unison and leveraged the weight of their bodies to inch the rope forward bit by bit. It would take more than an hour for them to bring in the net.

Mbacké Seck, director of the environmental organization Hann Baykeeper, grew up in Yarakh, the village where the fish market is situated. “When we were kids, the beach was full of fine white sand,” he says. “And there were so many fish that when there was a big wave, it could bring some of the fish to shore.”

Since 1960, Dakar’s population has grown from 444,000^8^ to nearly 2.5 million^9^ as people from the interior of Senegal flooded the city, fleeing drought and searching for new economic opportunities. Yarakh grew along with the rest of the city, as many of these new migrants became fishermen and built houses in the old village. There was no trash collection because the streets were too narrow to allow garbage trucks to enter, so residents threw their trash into the bay. There was no sewer system in this part of the city, either. Houses here still rely on latrines that owners have to pay to empty with special solid waste trucks. Many people do not want to pay the price, so they clean out the latrines themselves and dump their waste into a nearby canal or, often, directly into Hann Bay.^10^

Industries opened up along the bay’s shoreline—first just one or two, but now there are more than 100, according to the Senegalese government’s Directorate of Environment and Classified Establishments (DEEC). Some release soaps and salts from their production processes. The city’s main slaughterhouse discharges floods of blood, and a couple of food companies release water full of vegetable oil.

**Figure:**
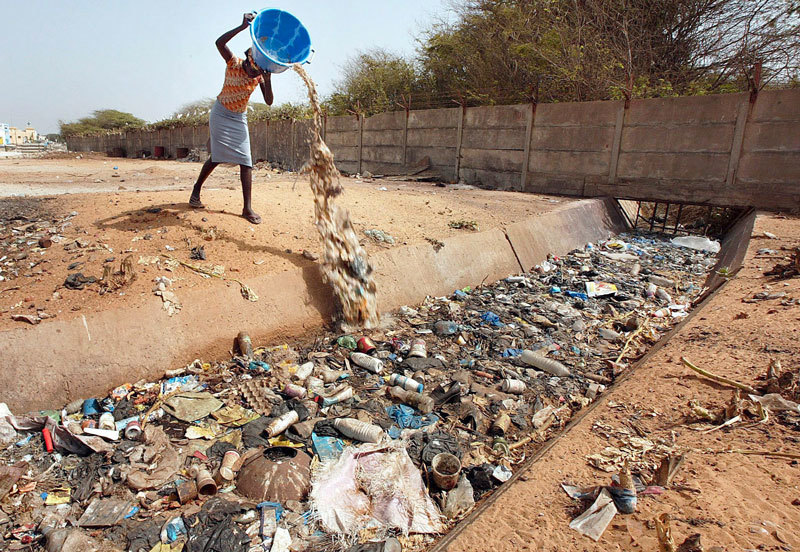
Dakar’s sanitation infrastructure hasn’t kept up with its rapid growth. As a result, some Hann Bay residents have little choice but to dump garbage and sanitary waste directly into the canals that feed into the bay. © Nic Bothma/epa/Corbis

“It’s not a chemical product, but pouring vegetable oil into the bay creates its own problems,” says El hadji Mamadou Sankharé, the bureau chief at DEEC in charge of making sure industries conform to environmental standards. “This kind of pollution, when you discharge it into the sea, in a way you are suffocating the aquatic environment and contributing to eutrophication.”

Soon, overfishing and pollution from all sides took a toll on the bay, says Mbacké Seck. “And suddenly, we realized that it was impossible to swim in the bay,” he says. “We noticed that there weren’t as many fish. And when the waves came in, there was just trash.”

## Cleaning Up the Bay

A project to clean up the bay has been in the planning phase for more than five years, with approximately $55 million worth of financing from the French Development Agency and the European Investment Bank.^11^ It is, Sankharé says, essentially an infrastructure project. The European Union is also financing an urban restructuring project for the villages of Yarakh and Petit Mbao.

Together the two projects will provide connection to the sewer system and wider roads to allow garbage trucks into the communities. The industrial factories along the shore will be connected to the sewer system as well. There will be a wastewater treatment station that will provide primary treatment (the removal of solids) before piping the wastewater nearly two miles offshore.

That wastewater treatment station will be built in the community of Petit Mbao, which is also home to the industrial zone. Primary treatment is certainly not enough for many of the industrial users, and the government is encouraging them to set up pretreatment protocols. Industrial companies are also supposed to pay for the pollution they create through a system of fees, which will be reorganized under the cleanup plan to a slightly different “polluter pays” plan that has not yet been finalized.

Senegal has signed the Stockholm Convention on Persistent Organic Pollutants, which bans the production, use, and unsafe disposal of substances including polychlorinated biphenyls (PCBs) and several pesticides.^12^ A number of other laws and codes regulate sanitation,^13^ wastewater management,^14^ air pollution,^15^ and discharges of mercury and lead.^16^

But Sankharé says his is a unit of one—he is the only person overseeing the environmental standards for every industrial company in the entire country. “Since we do not have the means to do our job as well as we should, it could happen that there might be discharges of PCBs or another chemical product that escape our vigilance,” he says.

**Figure:**
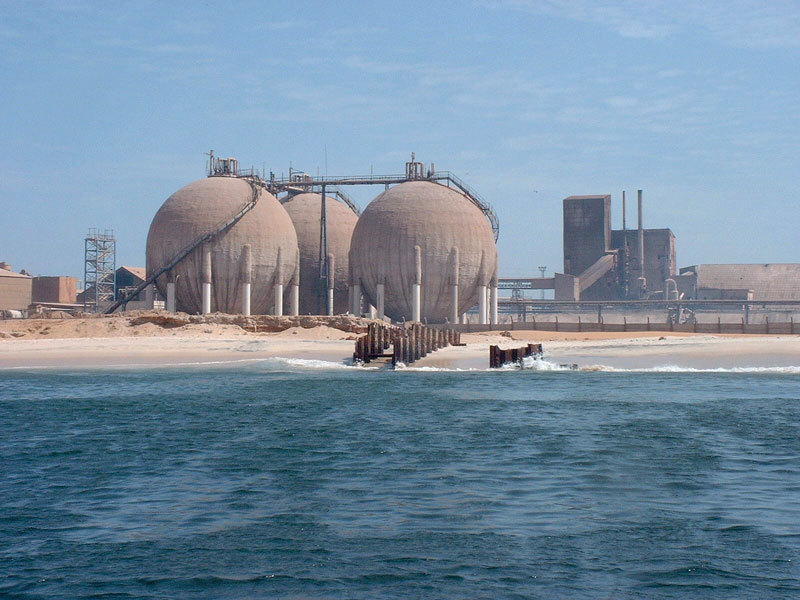
Other waste comes from more than 100 industrial facilities that line the shores of the bay, including this factory that processes phosphoric acid for fertilizer. Senegal has standards governing industrial emissions and waste management, but there is only one employee to enforce those standards for all the industries in the country. © Marc Bouvy

**Figure:**
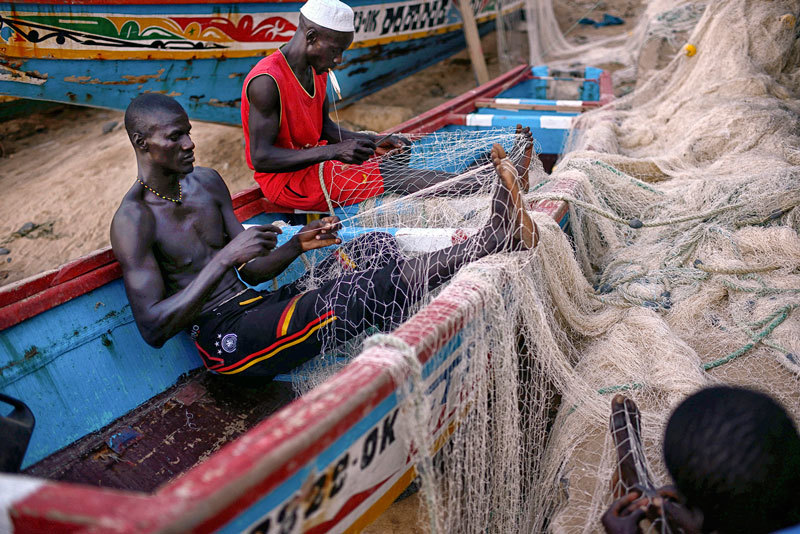
Fishing remains an important industry on Hann Bay, despite the pollution in the bay and the impact of overfishing. The human health effects of living and working near the water and eating contaminated food are unclear. © Samuel Aranda/Panos Pictures

## Filling Gaps

Momar Ndiaye, a professor in the chemistry department at Cheikh Anta Diop University (UCAD) in Dakar, is particularly interested in tracking contamination caused by polycyclic aromatic hydrocarbons (PAHs) from oil spills. Ndiaye conducted PhD research on the environmental impact of a large oil spill off the coast of Galicia in northern Spain, and now he is using mussels in Hann Bay to monitor the levels of contamination from Dakar’s oil refinery.^17^ Mussels live out their lives in one place, and because they are filter feeders, they absorb pollution in the water and are often used as pollution indicators.

“I saw that people gathered wild mussels from the rocks around the coast of Dakar, and they were eating them,” says Ndiaye. “So, I had the idea to do a study on the level of contamination of the mussels here.” He harvested mussels in the same locations for two consecutive years and found that, although the levels of PAHs were moderate in the mussels from Hann Bay and other parts of Dakar, they increased steadily over the study period.^17^ “In fact, the danger is that the pollution accumulates,” says Ndiaye. “And over a long period of time that could have an effect.”

That rationale also motivated researcher Cheikh Diop and his colleagues at UCAD’s Laboratory of Toxicology and Hydrology. “We saw that there was a real problem with the management of the coasts, especially related to industrial effluent,” he says. He and his colleagues started looking at heavy metals, too, especially lead and cadmium, which seemed to be particularly abundant in coastal Senegal. They sampled the sediment in sites around Dakar and along the coast and measured levels of contaminants in organisms including algae, mussels, shrimp, and several fish species.^18^^,^^19^^,^^20^

What they found was surprising. “When it came to heavy metal pollution in the sediment,” says Diop, “there was not a large difference between Hann Bay and Soumbédioune,” a bay on the other side of Dakar’s peninsula with no industries. But it makes sense. The canal that discharges effluent at Soumbédioune collects 60% of the whole city’s untreated wastewater. In fact, in all of Dakar, there is only one wastewater treatment station, in the village of Cambérène, and according to Sankharé, that facility processes only a fraction of the wastewater it receives. The rest just flows into the ocean.

**Figure:**
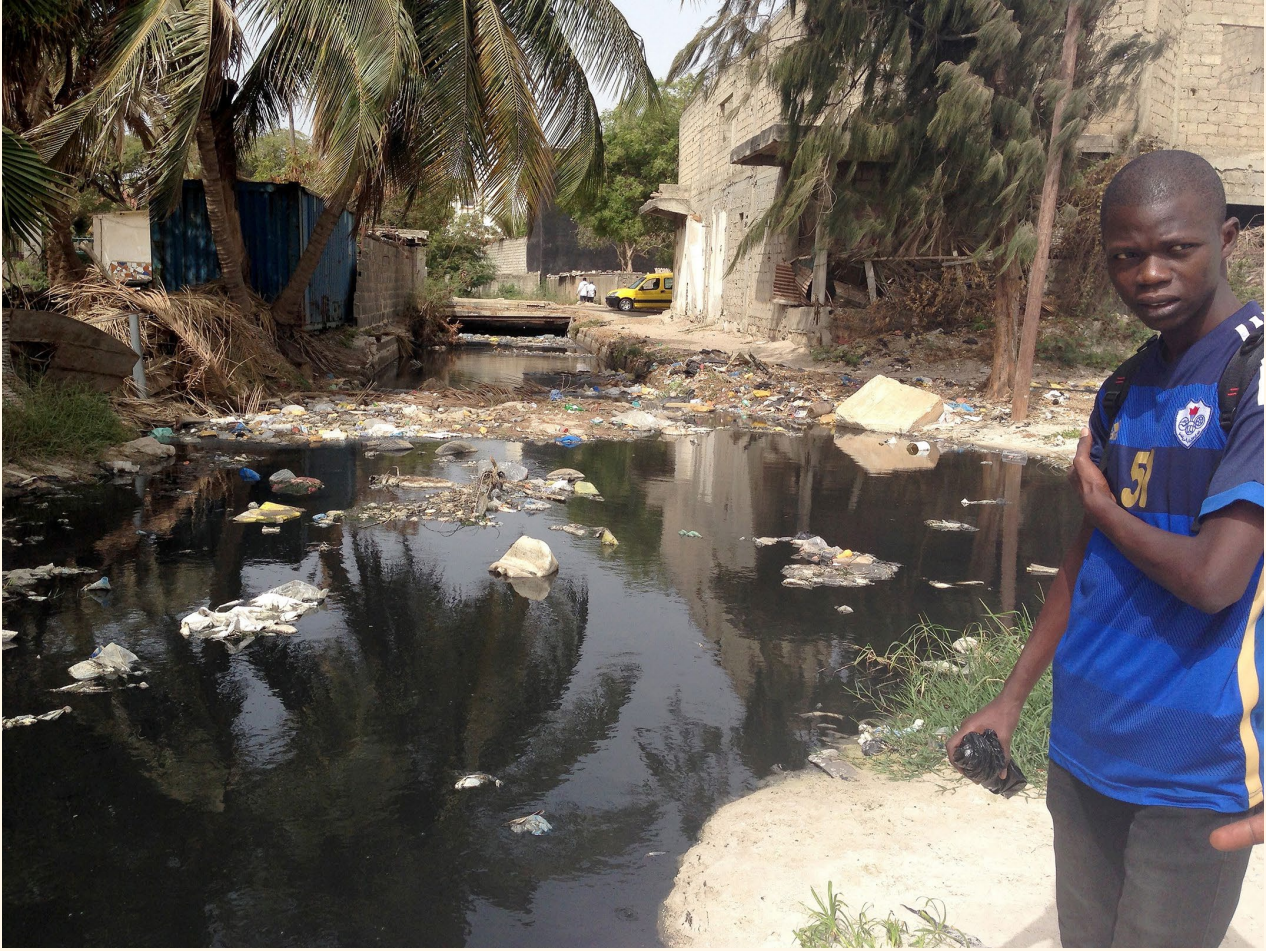
New infrastructure projects will provide sewer connections, roads that can accommodate garbage collection, and a new wastewater treatment plant to provide primary treatment before pumping effluent far offshore. These improvements won’t solve all of Hann Bay’s problems—but they’re a start. © Jori Lewis

These studies and others will help fill in the many gaps about the potential human health impacts of the pollution in Hann Bay. But the reality against which they are happening is that Hann Bay’s contamination is not exceptional; it’s typical of what is happening across Africa and the developing world, in places where infrastructure has not kept pace with development.^21^ And even as the Senegalese government starts to clean up Hann Bay, it has several others waiting.
